# Different states of synaptic vesicle priming explain target cell type–dependent differences in neurotransmitter release

**DOI:** 10.1073/pnas.2322550121

**Published:** 2024-04-24

**Authors:** Mohammad Aldahabi, Erwin Neher, Zoltan Nusser

**Affiliations:** ^a^Laboratory of Cellular Neurophysiology, Hungarian Research Network Institute of Experimental Medicine, Budapest 1083, Hungary; ^b^János Szentágothai School of Neurosciences, Semmelweis University, Budapest 1085, Hungary; ^c^Laboratory of Membrane Biophysics, Max Planck Institute for Multidisciplinary Sciences, 37077 Göttingen, Germany

**Keywords:** synaptic diversity, short-term plasticity, synaptic modeling, hippocampal interneurons, active zone

## Abstract

Pronounced differences in neurotransmitter release from a given presynaptic neuron, depending on the synaptic target, are among the most intriguing features of cortical networks. Hippocampal pyramidal cells (PCs) release glutamate with low probability to somatostatin expressing oriens-lacunosum-moleculare (O-LM) interneurons (INs), and the postsynaptic responses show robust short-term facilitation, whereas the release from the same presynaptic axons onto fast-spiking INs (FSINs) is ~10-fold higher and the excitatory postsynaptic currents (EPSCs) display depression. The mechanisms underlying these vastly different synaptic behaviors have not been conclusively identified. Here, we applied a combined functional, pharmacological, and modeling approach to address whether the main difference lies in the action potential-evoked fusion or else in upstream priming processes of synaptic vesicles (SVs). A sequential two-step SV priming model was fitted to the peak amplitudes of unitary EPSCs recorded in response to complex trains of presynaptic stimuli in acute hippocampal slices of adult mice. At PC–FSIN connections, the fusion probability (P_fusion_) of well-primed SVs is 0.6, and 44% of docked SVs are in a fusion-competent state. At PC–O-LM synapses, P_fusion_ is only 40% lower (0.36), whereas the fraction of well-primed SVs is 6.5-fold smaller. Pharmacological enhancement of fusion by 4-AP and priming by PDBU was recaptured by the model with a selective increase of P_fusion_ and the fraction of well-primed SVs, respectively. Our results demonstrate that the low fidelity of transmission at PC–O-LM synapses can be explained by a low occupancy of the release sites by well-primed SVs.

## Significance

Synaptic diversity is a key feature of neuronal networks. Diversity stabilizes network activity and increases computational capacity. A most intriguing example of synaptic diversity is the dependence of presynaptic release probability (Pv) and short-term plasticity on the postsynaptic target cell type. Two terminals of the same axon, separated by few microns only, release glutamate with an order of magnitude difference in Pv, depending on the type of the postsynaptic target cells. This was previously explained by differences in the probability with which fusion-competent vesicles are released by an action potential. Here, we test and confirm the hypothesis that a much larger contribution to diversity resides in vesicle priming, thus expanding the parameter space, which can contribute to synaptic diversity.

Three decades ago, it was shown that a single motor neuronal axon can form synapses with widely different short-term plasticity (STP) patterns depending on the targeted postsynaptic muscle type (1). Such postsynaptic target cell type–dependent differences in STP were also described in the rodent CNS (2–7). It was recognized that this functional synaptic heterogeneity increases the computational capacity of neuronal networks (8–10). For example, in the neocortex and hippocampus, pyramidal cells (PCs) innervate parvalbumin-expressing fast-spiking interneurons (FSINs) forming strong synapses with high synaptic vesicle (SV) release probability (Pv) with EPSCs exhibiting primarily short-term depression (STD). In contrast, when the same PC axons innervate somatostatin-expressing INs (e.g., mGluR1α-expressing oriens-lacunosum-moleculare (O-LM) cells in the hippocampus), the Pv is low and the postsynaptic responses display short-term facilitation (STF) (2–5, 11). The molecular mechanisms underlying differences in Pv and STP have been the subject of intensive research efforts in the past two decades, but no consistent picture has emerged (7, 12–16).

In view of the steep [Ca^2+^] dependence of SV release (17), the most obvious difference between PC–FSIN and PC–O-LM cell connections was considered to be a robust difference in the “effective” [Ca^2+^] that SVs “see” at their release sites (RSs). This could be either the consequence of a larger number (conductance) of voltage-gated Ca^2+^ channels (VGCC) or a shorter distance between these channels and the Ca^2+^ sensor of SV fusion. Almost two decades ago, Koester and Johnston (12) reported a smaller presynaptic [Ca^2+^] transient in cortical PC axon terminals that innervated bitufted (somatostatin expressing) interneurons (INs), compared to multipolar FSIN. A more recent study confirmed these findings in the hippocampus (16), but the difference in presynaptic [Ca^2+^] was only 30%. Despite this relatively small difference, a distinct coupling distance between the Ca^2+^ channels and SVs (7, 18, 19) may still explain the approximately 10-fold difference in Pv. To test this possibility, in a previous study (20), we directly measured the distance between Cav2.1 VGCC and Munc13-1 molecules [assumed to mark the SV release sites; (21)] using EM freeze-fracture replica labeling and found no significant difference between them at these two functionally distinct synapse populations. Furthermore, to compensate the slightly smaller [Ca^2+^] transients found in boutons innervating O-LM cells, we increased the Ca^2+^ influx into O-LM cell–innervating boutons to a level similar to that found in FSIN-innervating boutons with 5 µM 4-aminopyridine (4-AP). This manipulation increased the EPSC amplitude only by ~twofold, leaving a fivefold difference still unexplained (20). These results taken together indicate that differences in the fusion probability (P_fusion_) of fusion-competent (molecularly well-primed) SVs may not be the main reason for the 10-fold difference in Pv, where Pv is the function of P_fusion_ of fusion-competent SVs and the occupancy of RSs by such fusion-competent/well-primed SVs (Pv = P_fusion_ × P_occupancy_).

It has been shown (22–24) that heterogeneity of docked SVs with respect to their priming states at rest could explain distinct Pv. Likewise, the dynamics between different states during repetitive synaptic activity can cause pronounced differences in STP (22–32). This concept is captured by a recently published two-step priming model that assumes two sequential states of docking/priming prior to exocytosis: a loosely docked state (LS) followed by a fusion-competent tightly docked state (TS). Importantly, transitions between states are assumed to be reversible, resulting in a dynamic equilibrium at rest. Forward priming rates are Ca^2+^-sensitive, being enhanced by increases in intracellular [Ca^2+^]. Furthermore, only SVs of the TS pool can fuse upon arrival of an action potential (AP) (29, 30). According to this model, Pv is a function of P_fusion_ and the probability (P_TS_) that an SV is in the TS (Pv = P_fusion_ * P_TS_). Electron microscopy (EM) studies support the existence of morphologically distinct docking states of SVs and reveal essential roles of presynaptic proteins RIM, Munc13-1, CAPS, and SNAP25 (33–37).

Previously (20), we applied a pharmacological approach to probe the priming state of SVs at PC–FSIN and PC–O-LM synapses and found that the phorbol ester analogue phorbol dibutyrate (PDBU), which facilitates priming by activating Munc13s (38) and shortens the tethers between docked SVs and the active zone (AZ) membrane (33), produces a ~fivefold augmentation of unitary EPSCs at PC–O-LM connections, but only a 70% increase at PC–FSIN connections. Assuming that SVs at O-LM cell–innervating synapses are mainly in the LS state, this potent effect of PDBU is explained by a shift toward the TS state. Such a shift may be much smaller at SVs of FSIN-innervating synapses, a large fraction of which is already in TS at rest. Thus, differences in P_TS_ rather than P_fusion_ might be the main reason behind the differences in Pv at these two types of synaptic connection.

To test this hypothesis, here we carried out in vitro paired whole-cell recordings between hippocampal CA1 PCs and FSINs or O-LM cells and applied a set of simple and complex presynaptic stimulation protocols followed by mathematical modeling of the resulting EPSCs using the recently introduced sequential two-step priming model (29). Finally, we performed pharmacological manipulations of fusion and priming at PC–O-LM cell synapses and validated their selective effects on P_fusion_ and TS fraction, respectively.

## Results

### Short-Term Plasticity of CA1 PC to FSIN Connections.

To test the dynamic properties of release from CA1 PCs to FSINs, we performed simultaneous whole-cell patch-clamp recordings from CA1 PCs and INs in acute coronal slices obtained from the dorsal hippocampus of adult mice. Postsynaptic INs were visually identified based on the shape and location of their somata using DIC imaging. Their firing properties were tested using DC current injections of variable amplitudes. Once an IN was classified as FSIN (39) and a connected PC was found, recordings were performed within a 10 min window to avoid run-down of EPSCs (*Methods*). Consistent with a previous study (39), the amplitude of the first unitary EPSC was large (127.4 ± 109.7 pA, n = 106 pairs) and showed prominent cell-to-cell variability [coefficient of variation (CV): 0.87]. Although the majority of the unitary EPSCs had fast rise times (RT), some had 10 to 90% RT values >0.5 ms, which might indicate dendritic filtering of distally located synapses and potential space-clamp problems. To prevent complications arising from these factors, we subselected recordings in which the 10 to 90% RT of the averaged EPSC was <0.5 ms. These subselected well-clamped EPSCs had amplitudes of 160.5 ± 121.2 pA (n = 66 pairs) with a CV of 0.76.

**Fig. 1.**
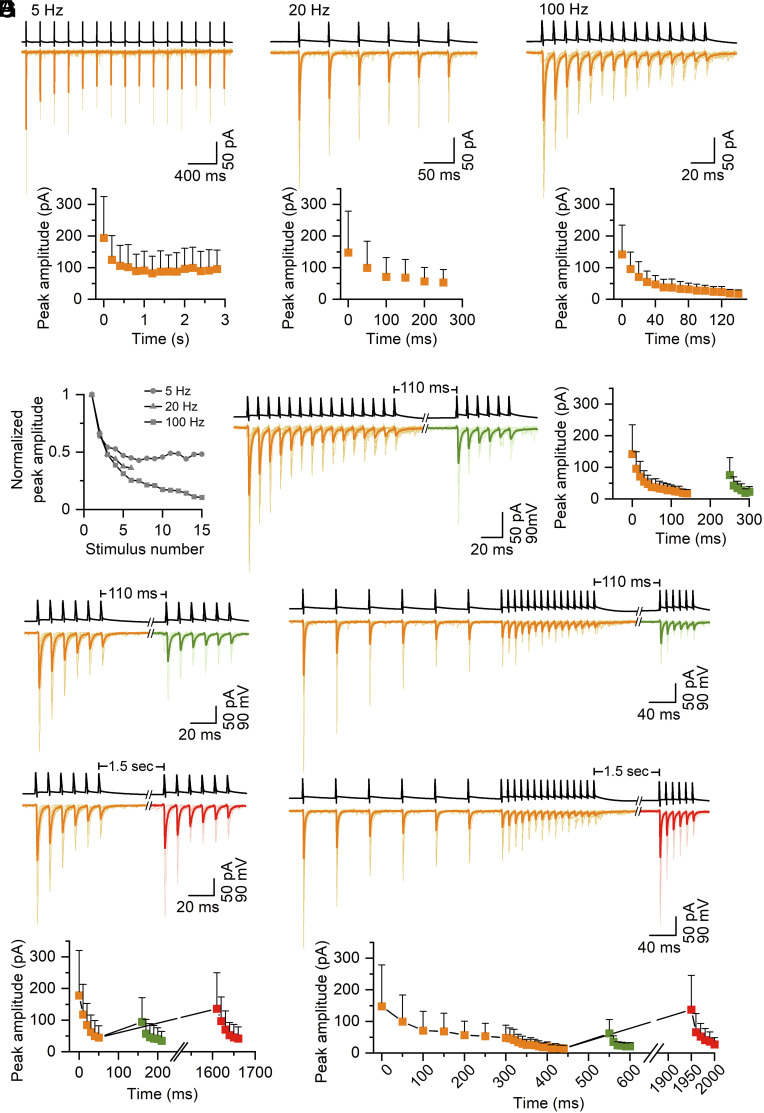
Short-term depression at PC−FSIN synapses. (*A*) *Top* panel. A train of 15 action potentials (APs) at 5 Hz in hippocampal CA1 PCs (black trace) evokes EPSCs (orange traces) in FSINs. Averaged EPSC traces are shown from individual pairs (light orange) and superimposed is the grand total average trace (GTA) of 12 recorded pairs (dark orange). *Bottom* panel. Evoked EPSC mean peak amplitudes are plotted as a function of time. (*B*) Same as (*A*) but 6 APs at 20 Hz (n = 20 for generating the GTA). (*C*) Same as (*A*) but 15 APs at 100 Hz (n = 21 for generating the GTA). (*D*) Normalized eEPSCs peak amplitudes from the GTA traces at 5, 20, and 100 Hz showing frequency dependence of steady-state depression. (*E*) 15 APs at 100 Hz followed by a short recovery train (6 AP at 100 Hz) after 110 ms. Examples of averaged eEPSC traces are shown from individual pairs (light orange, light green) as well as the GTA trace (dark orange and dark green, n = 21 pairs). eEPSC mean peak amplitudes are plotted vs. time (*Right*). (*F*) *Top* panel. Same as (*E*, *Left*) but in this protocol a short train (6 AP at 100 Hz) is followed by a short recovery train (6 AP, at 100 Hz) after 110 ms (n = 13, *Top*, green) or 1.5 s (the same 13 pairs, *Middle*, red). *Bottom* panel. EPSCs mean peak amplitudes are plotted vs. time (n = 13 pairs). In each pair, protocols with the two different recovery times were applied. The first 6 EPSC amplitude values are calculated from 20 traces in each pair, whereas the recovery 6 EPSC amplitudes from 10 and 10 traces. (*G*) Complex protocols composed of a preconditioning train (6 APs at 20 Hz) followed by a high-frequency long train (15 APs at 100 Hz) then a recovery short train after either 110 ms (6 APs at 100 Hz, n = 10, *Top*, green) or 1.5 s (n = 10, *Middle*, red). eEPSC mean peak amplitudes are plotted vs. time (*Bottom*). In the plot, the preconditioning and the 15 APs data were pooled together from the two protocols with different recovery times (n = 20 pairs).

To explore the dynamic properties of the FSIN-innervating synapses, we applied various stimulation protocols, testing both STF and STD, recoveries from facilitation/depression, and the effects of low-frequency conditioning on subsequent high-frequency trains. For the frequency dependence of release, we applied trains of presynaptic stimuli at 5, 20, and 100 Hz and recorded the postsynaptic responses (Fig. 1 *A–C*). Interestingly, the averaged paired-pulse ratio of the first two EPSCs (PPR_2/1_) was frequency independent (PPR_2/1_ at 5 Hz: 0.70 ± 0.18, at 20 Hz: 0.74 ± 0.3 and at 100 Hz: 0.74 ± 0.35), but the amplitudes of EPSCs at steady-state toward the end of the stimulus trains showed frequency-dependent depression (Fig. 1*D*; normalized amplitude from grand total average trace (GTA) at 5 Hz: 0.48, at 20 Hz: 0.37 and at 100 Hz: 0.14). We then tested the recovery of release at 110 ms after a long high-frequency train (15 APs at 100 Hz) and found that the relative amplitude of the first EPSC of the recovery pulses was 0.56 ± 0.23 of the first EPSC of the first train (n = 21), four times larger than that at the steady-state end of the preceding train (Fig. 1*E*). The recovery was very similar after a short (6 AP) 100 Hz train (relative amplitude of the first EPSC of the test train: 0.58 ± 0.37, n = 13; Fig. 1*F*). However, when the recovery time after the 6 AP train was increased to 1.5 s, the amplitude of the first EPSC recovered to 0.73 ± 0.21 of its original value (n = 13; Fig. 1*F*). We also tested these PC–FSIN synaptic connections with two “complex” protocols, in which a preconditioning train (6 APs at 20 Hz) was immediately followed by 15 APs at 100 Hz, which was followed by a short high-frequency test train (6 APs at 100 Hz) either at 110 ms or 1.5 s recovery times (Fig. 1*G*). The preconditioning 20 Hz train and the following 100 Hz train caused a moderate and robust depression, respectively. The recovery from depression depended on the interval between conditioning and test trains: The first EPSC of the test train recovered to 0.51 ± 0.20 of its original value after 110 ms and fully recovered after 1.5 s (1.16 ± 0.51; Fig. 1 *G*, *Bottom*).

### Modeling the PC–FSIN Synapses with a Sequential Two-Step Priming Model.

**Fig. 2.**
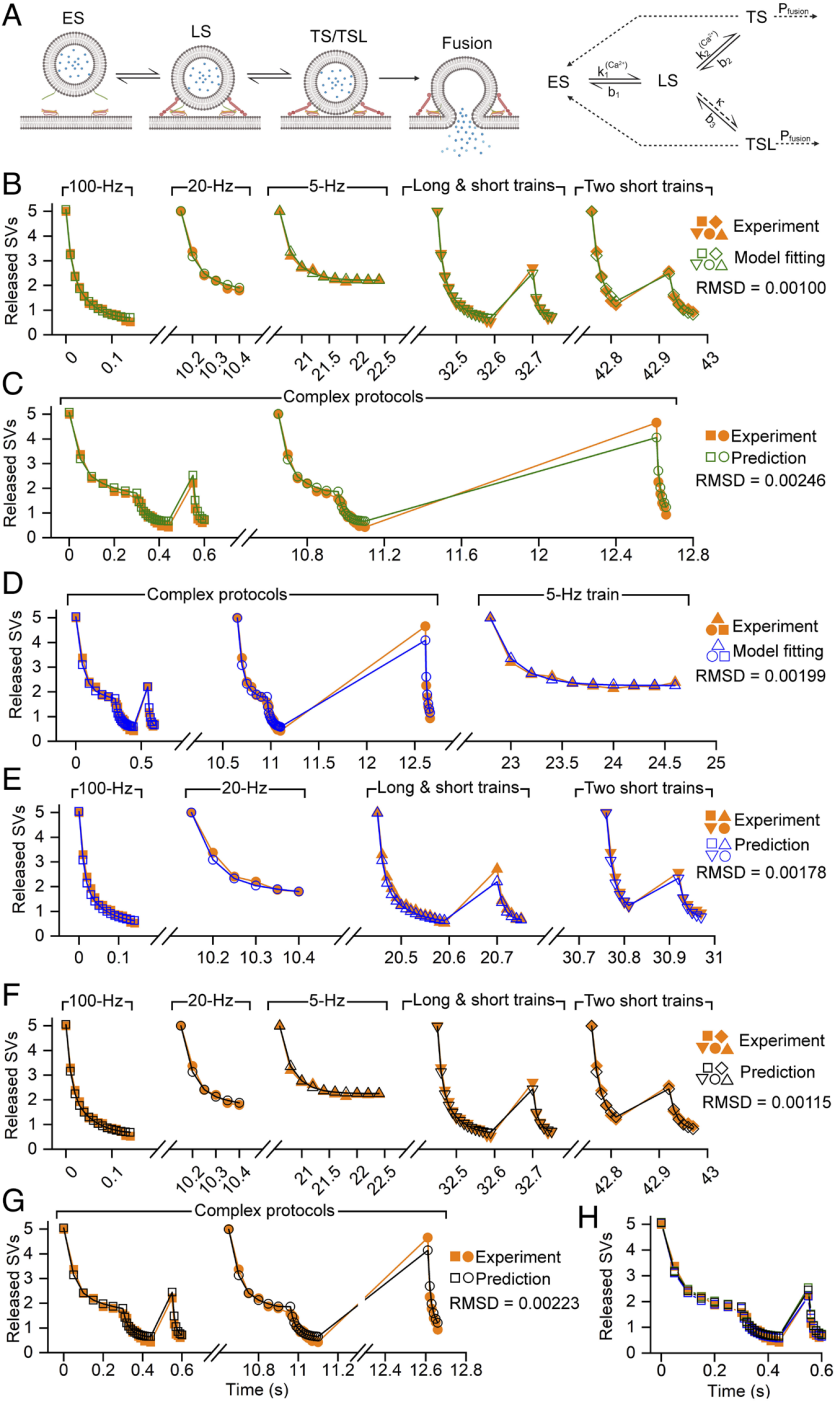
A sequential two-step priming model reproduces short-term depression patterns at PC−FSIN synapses. (*A*) *Left*: Schematic illustration of the sequential two-step priming model. Synaptic vesicles (SVs) can dock in empty docking sites (ES) and go through two sequential priming steps. In the first step, SVs are in a loosely docked state (LS) and are fusion incompetent from which they enter tightly docked states (TS or TSL) and become fusion competent. SVs from the TS and TSL states can fuse with the active zone membrane. *Right*: Kinetic scheme of state transitions between four states. A labile tightly docked state (TSL) needed to be introduced to describe robust facilitation at PC–O-LM synapses. b1, k1, b2, and k2 are rate constants, whereas k denotes the fraction of SVs that are transferred from the LS state to TSL after each action potential. The b3 is the decay time constant with which TSL returns to LS. b3 is approximately 50 times smaller than 1/b2. The model is adopted from ref. 29. (*B*) The sequential two-step priming and fusion model was fitted to PC−FSINs data obtained from five different protocols (shown in Fig. 1 *A–F*). rmsd, Root-mean-square deviation. (*C*) Experimental data of two complex protocols (Fig. 1*G*) and model prediction using the model parameters obtained in (*B*). (*D*) Same as (*B*) but the model fitting was performed on the two complex protocols and the 5 Hz train. (*E*) Experimental data and model predictions using the model parameters obtained in (*D*). (*F*) Experimental data and model predictions for the five simple protocols using the mean of the model parameters obtained in (*B*) and (*D*). (*G*) Same as (*F*) but for the two complex protocols. (*H*) Experimental data of one complex protocol superimposed onto model predictions from (*C*), (*D*), and (*G*). All experimental data shown are from the GTA traces. The X-axis indicates the time in seconds.

To estimate the proportion of well-primed SVs at FS–FSIN synapses, we turned to modeling of the EPSC amplitudes using the recently described sequential two-step priming model including a labile tightly docked SV state (TSL; Fig. 2*A* adopted from figure 1Ci in ref. 29). We simplified the model slightly by omitting the Ca^2+^ dependence of P_fusion_._._ We constrained the resting [Ca^2+^] to 50 nM with an increment of effective [Ca^2+^] following each AP of 110 nM (29). The remaining parameters were then fitted (see *Methods*; model parameters and terms related to the model are given and explained in *SI Appendix*, Table S1).

First, we performed parameter fitting simultaneously on data of five protocols, as shown in Fig. 2*B*: three simple trains (100, 20, and 5 Hz), the long train followed by a short one (15 + 6 APs at 100 Hz) and two short trains in sequence (6 + 6 APs at 100 Hz). Fig. 2*B* demonstrates the quality of the fit to these five protocols and Fig. 2*C* illustrates the model prediction with these parameters to the two complex protocols. Next, we performed the reverse sequence of analysis. We fitted the model parameters to the two complex protocols plus the 5 Hz steady-state train protocol (Fig. 2*D*) and tested the model prediction on the other four protocols (Fig. 2*E*). Visual inspection of the fit revealed an almost identical goodness of fit irrespective of whether the parameters were optimized on the five “simple” protocols (Fig. 2*B*) or on the complex trains (c.f. Fig. 2 *B* and *E*). Quantitatively, when the rmsd was calculated for the seven protocols, very similar values were obtained irrespective of whether the parameters were obtained from fitting the five simple protocols (0.00171) or the two complex protocols (0.00179). We then decided to calculate the mean of each parameter as obtained with the two methods and simulated all seven protocols using these mean values. This resulted in an rmsd (0.00165) that was somewhat smaller than those obtained separately (Fig. 2 *F–H*). Thus, our modeling demonstrates that similar model parameters are obtained when fitting is constrained to our complex protocols as compared to fitting data from the five simple protocols, offering the advantage of performing much less experiments for obtaining similarly constrained fits. This is of great importance, given the fragile nature of the synapses under study (time-dependent run-down after 10 min). Our model fitting/parameter optimization at PC–FSIN resulted in a P_fusion_ of 0.6 and a TS fraction (=TS/(TS+LS)) of 0.44, resulting in a Pv of 0.26 that is somewhat lower than that estimated with multiple probability fluctuation analysis (39). All model parameters together with an explanation of terms are provided in *SI Appendix*, Table S1.

### Short-Term Plasticity of CA1 PC–O-LM Cell Connections.

**Fig. 3.**
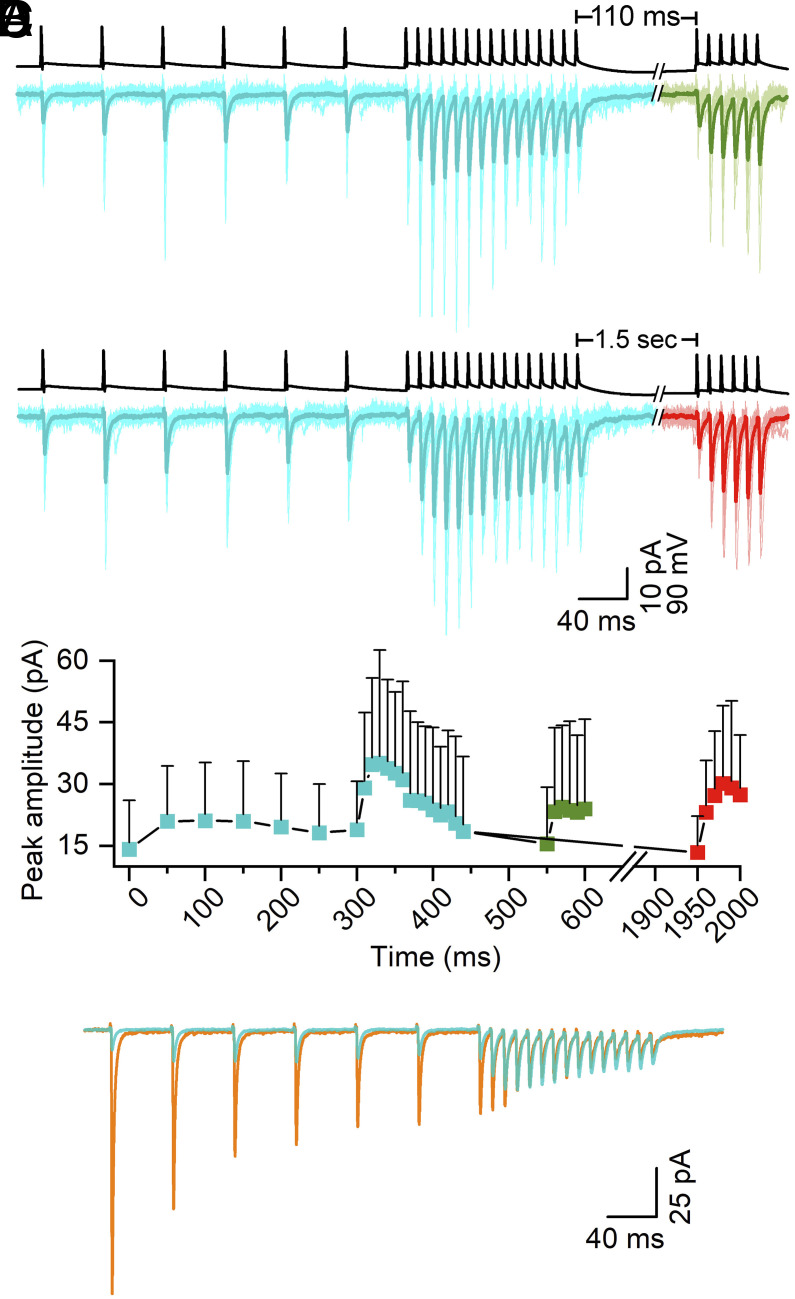
Short-term facilitation of PC–O-LM synapses. (*A*) Action potentials (APs) from hippocampal CA1 PCs (black trace) and evoked EPSCs (cyan and green) recorded in O-LM cells. Complex stimulation protocol composed of a preconditioning train (6 APs at 20 Hz), followed by a high-frequency long train (15 APs at 100 Hz), then a recovery short train after 110 ms (6 AP at 100 Hz, green). Averaged EPSCs are shown in individual pairs (light cyan or light green) with the grand total average (GTA, dark cyan and dark green, n = 30 pairs). (*B*) Same as (*A*) but with a recovery interval of 1.5 s (n = 20 pairs). The traces in the recovery period are shown in red. (*C*) eEPSC mean peak amplitudes are plotted vs. time; colors correspond to those of traces in (*B*) and (*C*). Data for the preconditioning and for the 15 AP-traces were pooled together from the two protocols (110 ms and 1.5 s recovery test durations, cyan points, n = 50 pairs). (*D*) Superimposed GTA traces from FSIN (orange) and O-LM (cyan) cells illustrate the dramatic difference in the short-term plasticity patterns.

To compare the above results with the properties of CA1 PC–O-LM cell synapses, we performed dual whole-cell recordings in acute slices of transgenic mice in which td-Tomato is expressed in O-LM INs (see *Methods* and ref. 20). The firing properties of fluorescent INs were examined with DC current injections of different amplitudes. The recorded INs were filled with biocytin and their morphological identity was verified post hoc. The STP of release at PC–O-LM cell connections was tested using the two complex protocols only. Consistent with the results of previous studies (20, 40, 41), the amplitudes of first EPSC of trains were small (14.2 ± 11.9 pA, n = 50 pairs), failure rates were high, and some connections had only failures for the first AP (6 out of 50). The amplitude of EPSCs slightly increased (facilitated) during the 20 Hz preconditioning train and more dramatically during the first few APs of the 100 Hz train episode (Fig. 3 *A–C*). The normalized amplitude of the first EPSC of recovery test train after 110 ms was 1.50 ± 1.66 (normalized to the first EPSC of the preconditioning train, n = 26), whereas EPSCs fully recovered (0.95 ± 0.54, n = 18 pairs) after 1.5 s (Fig. 3*C*). Fig. 3*D* illustrates the superimposed GTA traces of the PC–O-LM and PC–FSIN connections, demonstrating that the >10-fold difference in the first EPSC amplitude disappears during stimulation and that after the ninth AP both synapses can maintain transmission during high-frequency presynaptic activity roughly equally well.

### Modeling Transmission at PC–O-LM Cell Connections Suggests a Very Small Fraction of Tightly Docked Vesicles.

**Fig. 4.**
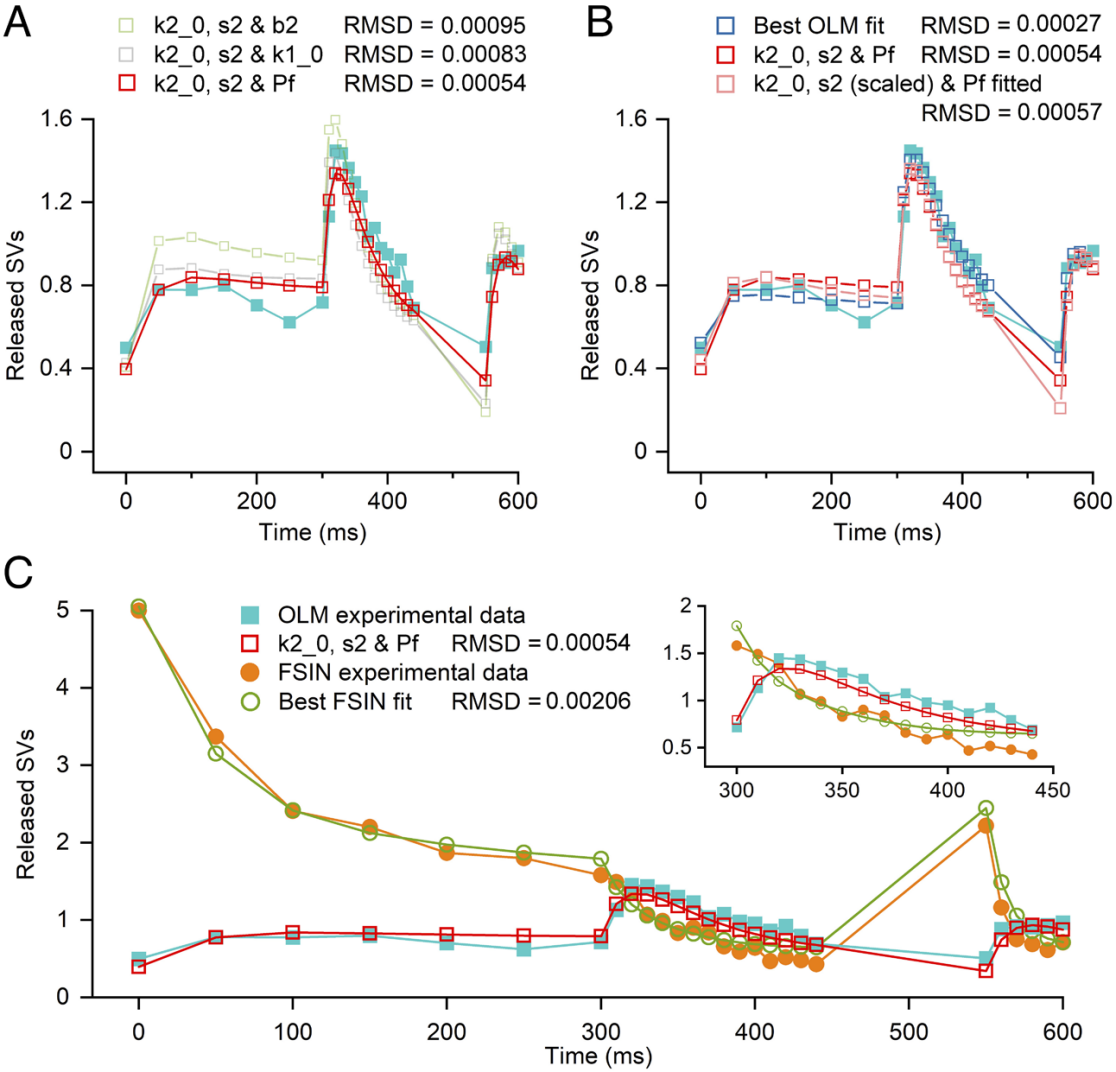
Changing model parameters related to the second SV priming step plus P_fusion_ are sufficient to change PC−FSIN-like release to PC−O-LM-like release dynamics. (*A*) All parameters of the two-step SV priming model were obtained from the best fit to the PC−FSIN data (Fig. 2 *E* and *F*), and three parameters were fitted to the PC–O-LM experimental data. Qualitatively, all three illustrated simulations describe the STP pattern, but fitting k2_0, s2, and Pf reproduces the data with the smallest error (red). (*B*) The best O-LM fit (blue), k2_0, s2, and Pf fit [as shown in panel (*A*); red] and fit in which k2_0 and s2 were constrained to scale together with one scaling factor (light red) are shown. (*C*) Experimental data and best model fit for PC–FSIN (Fig. 2*G*) and the k2_0, s2 and Pf fit for PC–O-LM (*A*) are superimposed for direct comparison using the same complex protocol. The *Inset* shows the episode of 100 Hz stimulation at better resolution.

Next, we aimed to determine the key model parameters that are responsible for the differences in the functional properties of PC–FSIN and PC–O-LM cell connection. We started with the parameter values optimized for the PC–FSIN connections (Fig. 2 and *SI Appendix*, Table S1) and varied parameters one by one to see whether the model predictions fit the PC–O-LM cell data. First, we allowed only a single, then two then three parameters to be changed simultaneously (*Methods*) and found progressively better and better fits. When k2_0, s2, and P_fusion_ were simultaneously optimized, the model qualitatively described the initial small facilitation and depression, followed by the large facilitation and depression during the high-frequency EPSC train (Fig. 4*A*). Recognizing that k2_0 and s2 together determine the Ca^2+^ dependence of k2, we also introduced a scaling factor for k2_0 and s2 of the FSIN fit and optimized this scaling factor together with P_fusion_. This resulted in an almost identical goodness of fit to that obtained when the three parameters were fitted separately (Fig. 4*B*). Finally, we also allowed all model parameters to be fitted, resulting in a slight improvement in the overall fit (Fig. 4*B*). Fig. 4*C* illustrates the PC–FSIN experimental data and superimposed best model fits as well as the PC–O-LM cell data with the model fit for which only three parameters were altered.

We then examined the effects of changing these three model parameters and found that a >10-fold reduction of k2_0 and s2 resulted in a dramatic 6.5-fold reduction of the proportion of tightly docked SVs (TS fraction = 0.07 vs. 0.44 for the FSINs), whereas the reduction in P_fusion_ was only 40% (from 0.60 to 0.36; *SI Appendix*, Table S1). These results demonstrate that the sequential two-step priming model is capable of describing the release dynamics of both PC–FSIN and PC–O-LM synapses and that altering only three parameters can convert a depressing into a facilitating synaptic “phenotype.” Disregarding small deficits, one could even switch between the two functional “phenotypes” by just changing two parameters: P_fusion_ and the scaling factor (steepness of the Ca^2+^ dependence of k2).

### Selective Pharmacological Manipulation of P_fusion_ and the Proportion of Tightly Docked SVs.

In the experiments described so far, we reached our conclusions by fitting the sequential two–step priming model to our experimental data obtained from PC–FSIN and PC–O-LM connections and drew our biological conclusions based on model parameters of P_fusion_ and the TS fraction. In our final set of experiments, we aimed to validate our approach by using selective pharmacological manipulations targeting either P_fusion_ or the TS pool size. The K^+^ channel blocker 4-AP broadens APs and increases AP-evoked [Ca^2+^] transients (20) and consequently increases P_fusion_. The phorbol ester analog PDBU increases release by facilitating priming of SVs through the positive modulation of Munc13s without effecting AP-evoked [Ca^2+^] transients (20), hence, PDBU should not affect P_fusion_. Thus, we recorded the effects of these drugs on PC–O-LM cell connections and performed model optimization to investigate which model parameters are altered in the presence of these two drugs.

**Fig. 5.**
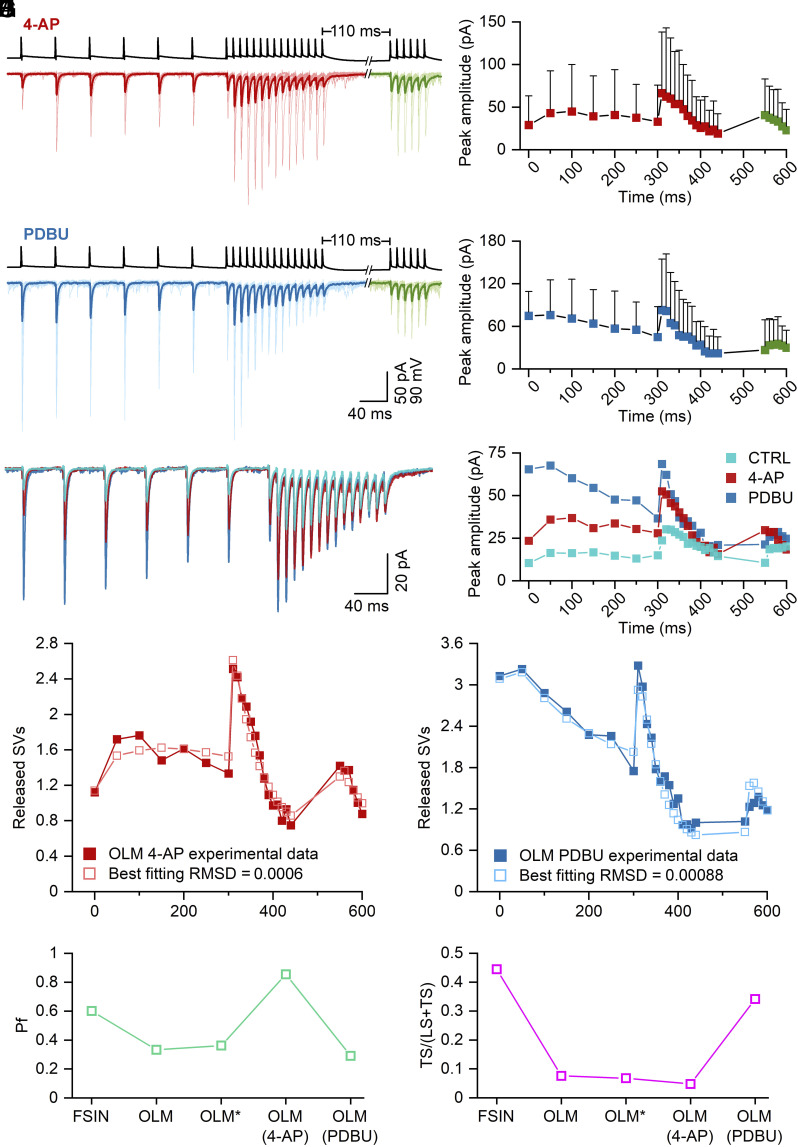
Selective pharmacological manipulation of SV fusion and priming. (*A*) Postsynaptic responses to a complex protocol composed of a preconditioning train (6 APs at 20 Hz), followed by a high-frequency long train (15 APs at 100 Hz), then a recovery short train after 110 ms (6 AP at 100 Hz) recorded in O-LM cells upon the stimulation of a CA1 PCs (black trace) in the presence of 5 µM 4-AP. Averaged EPSCs are shown in individual pairs (light red and light green) with the grand total average trace (GTA, dark red and dark green, n = 23 pairs) superimposed. (*B*) eEPSCs mean peak amplitudes in 4-AP are plotted vs. time; colors correspond to traces in A (n = 23). (*C*) Same as (*A*), but in 1 µM PDBU. Averaged EPSCs are shown in individual pairs (light blue and light green) with GTA (dark blue and dark green, n = 15 pairs) superimposed. (*D*) eEPSC mean peak amplitudes in PDBU are plotted vs. time; colors correspond to traces in C (n = 15). (*E*) Superimposed GTA traces from O-LM cells in control (cyan), in 4-AP (red), and in PDBU (blue) illustrate the effect of these drugs. (*F*) eEPSC mean peak amplitudes from GTA traces are plotted vs. time; colors correspond to traces from (*E*). (*G* and *H*) The sequential two-step SV priming and fusion model was fitted to PC–O-LM data in 4-AP (*G*) and in PDBU (*H*). (*I*) Best fit values of P_fusion_ at PC−FSIN and PC– O-LM connections in the absence or presence of 4-AP or PDBU. Best fit for FSIN, O-LM, 4-AP, and PDBU is shown. O-LM* denotes to P_fusion_ value when only k2_0, s2, and P_f_ parameters were fitted to O-LM data. (*J*) Same as (*I*) but for the TS fraction.

In the presence of 5 µM 4-AP, the amplitude of the first EPSC of the preconditioning train was 29.1 ± 34.2 pA (n = 23 pairs), which is approximately twofold larger than that in control ACSF (Fig. 5). The pattern of postsynaptic responses remained very similar to that found in control recordings: There was a small facilitation during the preconditioning train and a robust facilitation–depression during the 100 Hz train. However, the recovery at 110 ms was less pronounced; the normalized response of the first recovery pulse was 1.74 ± 1.02 (relative to the first EPSC of the preconditioning train), which was larger than that found in control (1.50 ± 1.66; Fig. 5 *A*, *B*, and *F*).

Postsynaptic responses in the presence of 1 µM PDBU were drastically different. The amplitude of the first EPSC of the preconditioning train was >sixfold larger than that recorded in ACSF (74.9 ± 86.3 pA, n = 15; Fig. 5 *C* and *D*) and the EPSCs during the preconditioning train displayed mainly depression rather than facilitation. At the beginning of the 100 Hz train, the facilitation was also smaller than that in control (2.0 ± 1.8-fold vs. 3.1 ± 2.5 in control; Fig. 5 *E* and *F*). These pharmacological experiments confirm our previous results (20) showing a much larger effect of PDBU than that of 4-AP on the amplitude of the first EPSC of the train at PC–O-LM cell synapses.

We then fitted our model to the data obtained in 4-AP and PDBU with the following constraints. In our previous study (20), we demonstrated that 5 µM 4-AP resulted in a ~50% increase in the AP-evoked Ca^2+^ influx in axon terminals. Thus, we increased the AP-induced [Ca^2+^] increment from 110 nM to 168 nM in our model. The resting [Ca^2+^] was fixed to 50 nM. We then optimized all other parameters and obtained a fit that qualitatively described well the STP pattern of the data under 4-AP (Fig. 5*G*). Because our experimental data demonstrated that the application of PDBU did not change the AP-evoked Ca^2+^ influx into hippocampal PC axon terminals (20), we constrained the parameters describing AP-evoked [Ca^2+^] transients to those of controls and fitted the rest of the parameters to the PDBU data. The model with all other parameters as freely variable ones also produced a good fit to the data (Fig. 5*H*), describing well the pattern of the plasticity during the complex protocol. We then looked at how these drugs affected the two key functional parameters. 4-AP increased the P_fusion_ by 2.6-fold (to 0.85) without any major change in the proportion of SVs in the TS state (TS fraction: 0.05 in 4-AP vs. 0.08 in control; Fig. 5*I*). In contrast, the best fit to the PDBU data resulted in an almost identical P_fusion_ to that obtained in control (0.29 vs. 0.33) with a 4.5-fold increase in the TS fraction (from 0.08 to 0.34; Fig. 5*J*; see all model parameters in *SI Appendix*, Table S1). These results verify that the selective pharmacological modification of priming and fusion altered model parameters that influence TS fraction and P_fusion_, respectively, in a predictable manner.

## Discussion

In the present study, we used a combined experimental and modeling approach to investigate the mechanisms of postsynaptic target cell type–dependent differences in release and STP in hippocampal circuits. We used a recent sequential two-step priming model (29) to simulate experimental data obtained from paired recordings between CA1 PC and FSINs or O-LM cells using several presynaptic activity protocols. Our results revealed that this model accurately describes all our data obtained from both IN types under control conditions and in the presence of two drugs (4-AP and PDBU). Our results indicate that the main difference underlying the distinct Pv of these synaptic connections lies in a robust difference in the fraction of well-primed SVs (TS fraction) rather than the fusion probability of such SVs (P_fusion_) (see *SI Appendix*, Table S1 for a description of model terms and parameter values).

To transform the sequential two-step priming model that describes the PC–FSIN transmission to that representing PC–O-LM synapses, only three parameters needed to be changed: P_fusion_, k2_0, and s2. While P_fusion_ had to be decreased only moderately (by 40%), parameters associated with the second priming step (its rate constant at rest, k2_0, and the steepness of its Ca^2+^ dependence, s2) needed to be decreased by over 10-fold, resulting in a >sixfold reduction in TS fraction. All other model parameters could take on identical values for these two types of synapses. Furthermore, if k2_0 and s2 were fitted simultaneously with a joint scaling factor, the rmsd value was only slightly larger than that obtained by fitting them separately (0.00057 vs. 0.00054). Thus, changing only two parameters (P_fusion_ and the scaling factor) was also sufficient to convert the model from PC–FSIN synapses into an almost optimal model for PC – O-LM synapses.

P_fusion_ is controlled by the number/density, conductance, and open probability of VGCCs in the AZ. In addition, P_fusion_ is also determined by the Ca^2+^ sensitivity of Ca^2+^ sensors on SVs and the distance between the VGCCs and Ca^2+^ sensors (19, 42–47). Our modeling predicted a less than twofold difference in P_fusion_ at PC–FSIN (0.6) vs. PC–O-LM (0.36) synapses. This difference in P_fusion_ might be explained by the 40% larger AP-evoked [Ca^2+^] transients measured at PC–FSIN boutons (12, 16, 20). Indeed, 5 µM 4-AP, which increased the Ca^2+^ influx by ~40% at PC–O-LM connections resulted in a twofold increase in EPSC amplitude and P_fusion_ (20). What could be the reason for the 40% larger presynaptic Ca^2+^ influx at PC–FSIN connections? EM freeze-fracture replica immunolabeling demonstrated a 20% larger density of Cav2.1 in parvalbumin-positive dendrite-innervating AZs (20). The remaining ~20% difference might originate from a smaller conductance or a lower open probability of VGCCs at PC–O-LM cell synapses, which could be achieved by an mGluR-mediated mechanism (48). To estimate the coupling distance between VGCCs and Ca^2+^ sensors, we have previously performed EM freeze-fracture replica immunogold labeling of Cav2.1 VGCCs and Munc13-1 as a molecular marker of the RS. Our data demonstrated no significant difference in coupling distances at these two connection types (20). The Ca^2+^ sensor of fusion is very likely synaptotagmin-1 in both synapses, suggesting a similar Ca^2+^ sensitivity of fusion. All these data provide evidence for a ~20% larger Cav2.1 VGCC density, a 40% larger [Ca^2+^] transient in PC boutons innervating FSINs, which could fully explain the <twofold difference in P_fusion_ between these synapses.

In contrast to the modest difference in P_fusion_, our modeling indicates a robust difference between PC–FSIN and PC–O-LM synapses in the fraction of SVs that are in a well-primed state at rest (0.07 vs. 0.44). A small fraction of docked well-primed SVs explaining low Pv was proposed a long time ago and was supported by experimental data (22–24). In addition, it was also put forward that dynamic change in the fraction of well-primed SVs during repetitive presynaptic activity could underlie STP (22–32). A recent study by Lin et al. (29) demonstrated that differences in resting TS fraction underlie heterogeneity in Pv among individual calyx of Held synapses. Lin et al. (29) also demonstrated that diversity in P_fusion_ is not required to explain heterogeneity in STP at this synapse. The fact that the proportion of well-primed SVs shows large synapse-to-synapse heterogeneity offers the possibility that it might be the consequence of the specific modulation at a given synapse by long-term plastic mechanisms. Indeed, it was shown that after presynaptic LTP induction the fraction of well-primed SVs at neocortical L5 PC synapses is increased (49). Likewise, at parallel fiber to Purkinje cell synapses of the cerebellum, LTP is associated with an increase in the readily releasable pool of SVs (50).

Our modeling also provides an explanation of STF at PC–O-LM synapses. At this synapse, our model prediction is that most SVs are in the LS state at rest (0.93), which results in a low TS faction (0.07) and therefore a low Pv (~0.025). During high-frequency repetitive stimulation, SVs shift from the LS to TS state in an accelerated manner due to the Ca^2+^ sensitivity (s2) of the forward rate constant k2, resulting in STF at frequencies above 10 Hz. This mechanism, however, is not sufficient to explain the full extent of STF at high frequencies. Rather, in agreement with Lin et al. (29), we have to assume that approximately 20% of SVs that reside in the LS state are transferred to a labile TS state (TSL) following each AP, from which release can occur with a probability of P_fusion_. In contrast to TS, this state is labile, returning to LS within ~40 ms (b3), which is >25 times faster than the backward rate constant from the TS state (b2). Therefore, TSL does not contribute to STF at low stimulus frequencies (when the interstimulus interval is >40 ms), but it has a robust role in STF at high (e.g., at gamma frequencies) stimulus frequencies. Interestingly, our model without incorporating a Ca^2+^-dependent increase in P_fusion_ can fully explain one of the most robust known STF of cortical networks.

What are the structural correlates of SVs in LS and TS? We interpret the two states of our model in terms of tight and loose docking in view of recent cryo-EM studies suggesting that SVs with distances shorter than 5 nm from the plasma membrane might constitute well-primed SVs and correspond to the TS state in our model, whereas those at 5 to 10 nm from the AZ membrane could form the LS pool (33). Compared to cryo-EM, when brain tissue is chemically fixed, the corresponding SV–AZ membrane distances are shorter because of the membrane perturbation due to the heavy metal staining and dehydration. Therefore, it is possible that, following chemical fixation, those SVs that are in direct contact with the AZ membrane correspond to SVs in the TS state, and those that are a short distance from the AZ (1 to 5 nm) correspond to SVs in the LS state (30, 33, 34, 51). Results of these EM studies as well as from this study suggest that there should be a large difference in the number of SVs that are in direct contact with the AZ plasma membrane between PC–FSIN and PC–O-LM synapses. However, in a previous work, we directly tested this hypothesis using EM tomography of chemically fixed hippocampal slices after immersion fixation or after high-pressure freezing and found similar densities of docked SVs at FSIN and O-LM cell-targeting PC AZs (~135 SV/µm^2^) (20). Therefore, it is an open question, whether the postulated functional states of the model actually reflect these two morphologically defined states or else represent any other difference in the state of the release machinery. It is intriguing, though, that Munc13-1, a priming protein with Ca^2+^- and DAG-dependent regulatory sites, was postulated to exist in two conformations with different orientations relative to the plasma membrane (52). Our results might suggest that the physical docking does not necessarily mean molecular maturation/priming of SVs. Future experiments with well-conceived genetic modifications and EM techniques will be needed to resolve these discrepancies.

## Methods

### Animals.

Fifty-six adult (P48–94) male and female transgenic mice were used (Chrna2-Cre)OE25Gsat/Mmucd, [RRID:MMRRC_036502-UCD, on C57BL/6 J background (53)] and crossed with reporter line Ai9 or Ai14 [Gt(ROSA)26Sor_CAG/LSL_tdTomato]. The animals were housed in the vivarium of the Institute of Experimental Medicine in a normal 12 h/12 h light/dark cycle and had access to water and food ad libitum. All experiments were carried out in accordance with the Hungarian Act of Animal Care and Experimentation 40/2013 (II.14) and with the ethical guidelines of the Institute of Experimental Medicine Protection of Research Subjects Committee.

### Slice Preparation.

Mice were stably anesthetized with a ketamine, xylazine, pypolphene cocktail (0.625, 6.25, 1.25 mg/mL respectively, 10 µL/g body weight) then decapitated, the brain was quickly removed and placed into an ice-cold cutting solution containing the following (in mM): sucrose, 205.2; KCl, 2.5; NaHCO_3_, 26; CaCl_2_, 0.5; MgCl_2_, 5; NaH_2_PO_4_, 1.25; and glucose, 10, saturated with 95% O_2_ and 5% CO_2_. Then, 250 µm thick coronal slices were cut from the dorsal part of the hippocampus using a Vibratome (Leica VT1200S) and were incubated in a submerged-type holding chamber in ACSF containing the following (in mM): NaCl, 126; KCl, 2.5; NaHCO_3_, 26; CaCl_2_, 2; MgCl_2_, 2; NaH_2_PO_4_, 1.25; and glucose, 10, saturated with 95% O_2_ and 5% CO_2_, pH = 7.2 to 7.4, at 36 °C for 30 min, and were then kept at 22 to 24 °C.

### Electrophysiological Recordings.

All paired recordings were performed at 32 to 33 °C up to 6 h after slicing in ACSF supplemented with 2 µM AM251 to block presynaptic CB1 receptors and 0.35 mM γDGG to prevent AMPA receptors saturation. Cells were visualized with infrared differential interference contrast (DIC) imaging on a Nikon Eclipse FN1 microscope with a 40x water immersion objective (NA = 0.8). CA1 PCs were identified based on their position and morphology. O-LM INs were identified in the stratum oriens of the CA1 region by tdTomato expression, somatic morphology, and the membrane voltage responses upon de- or hyperpolarizing current injections (600 ms, from −250 to 800 pA with 100 pA steps). FSINs were identified using their position, morphology, and their membrane voltage responses upon de- or hyperpolarizing current injections (600 ms, from −250 to 800 pA with 100 pA steps). Patch pipettes (resistance 4 to 6 MΩ) were pulled from thick-walled borosilicate glass capillaries with an inner filament. For the interneurons, intracellular solution contained the following (in mM): K-gluconate, 130; KCl, 5; MgCl_2_, 2; EGTA, 0.05; creatine phosphate, 10; HEPES, 10; ATP, 2; GTP, 1; and biocytin, 7. For the PCs, intracellular solution contained the following (in mM): K-gluconate, 97.4; KCl, 43.5; MgCl_2_, 1.7; NaCl, 1.8; EGTA, 0.05; creatine phosphate, 10; HEPES, 10; ATP, 2; GTP,0.4; biocytin, 7 and 10 mM glutamate, pH = 7.25; 290 to 305 mOsm. Paired whole-cell recordings were performed while the PCs were held in current-clamp mode at −65 mV (with a maximum of ±100 pA DC current). Postsynaptic INs were held at −65 mV in voltage-clamp mode (with a maximum of ±200 pA DC current) with access resistance below 20 MΩ using a dual-channel amplifier (MultiClamp 700B; Axon Instruments). APs were evoked in PCs with 1.5 ms long depolarizing current pulses (2.3 nA). Evoked EPSCs were recorded for PC–FSINs pairs using six different protocols: 1) 15 APs at 5 Hz; 2) 15 APs at 100 Hz followed by 6-APs recovery test train after 110 ms at 100 Hz; 3) 6 APs at 100 Hz followed by a 6-APs recovery test train at 100 Hz after 110 ms; 4) 6 APs at 100 Hz followed by a 6-APs recovery test train at 100 Hz after 1.5 s recovery test period; 5) 6-APs preconditioning train at 20 Hz followed by 15-APs train at 100 Hz then 6-APs recovery test train at 100 Hz after 110 ms; and 6) 6-APs preconditioning train at 20 Hz followed by 15-APs train at 100 Hz then 6-APs recovery test train at 100 Hz after 1.5 s recovery test period. For the PC–O-LM pairs, only the last two protocols were recorded. Recording from a given pair was restricted to 10 min to avoid rundown of postsynaptic responses. Sixty-second intertrace intervals were kept except for the third and fourth protocols where the 30 s intertrace interval was used. INs with an increased access resistance (>25%) during the recording period were excluded from analysis. Data were filtered at 3 to 4 kHz (Bessel filter), digitized online at 50 kHz, recorded, and analyzed using Clampfit 10.7 (Molecular Devices). The peak amplitudes, 10 to 90% RTs, and areas under the curves were calculated in Clampfit. Likewise, average traces and their 10 to 90% RTs were calculated from 10 or 20 traces in Clampfit for each PC-FSIN pair. Pairs with RTs <0.5 ms of the first EPSC of the train were subselected and further used in the study.

To perform recordings after a fixed duration of drug application while maintaining short (10 min) whole-cell recording time to minimize EPSC rundown, we modified our experimental protocol. Fluorescent O-LM INs were selected and patched in acute coronal slices of the dorsal hippocampus of transgenic mice and synaptically connected presynaptic PCs were searched. Once a connection was established, the patch pipette from the PC was withdrawn, and the drug was perfused onto the slice. Ten minutes later, the PC was repatched and EPSCs to the complex protocol with 110 ms recovery were recorded for 10 min only. We have previously found that 5 µM 4-AP increases Ca^2+^ influx at PC boutons targeting mGluR1α expressing INs to the same level that was found at PC boutons targeting parvalbumin expressing INs (20).

### Modeling Short-Term Plasticity.

The sequential two-step priming model (29) was implemented in Berkeley Madonna [version 10.4; (54)]. Michaelis–Menten-like saturation for k1_0 in response to [Ca^2+^] was implemented, but the Ca^2+^ dependence of P_fusion_, as described in ref. 29, was omitted. In addition to LS and TS, a “Labile Tight State” (TSL), which contributes to release at high frequencies, was taken into account according to ref. 29. Parameter fitting was done by the algorithm of the software (Euler’s method). Unless otherwise stated, the resting [Ca^2+^] was constrained to 50 nM, and the increment of effective [Ca^2+^] following each AP was constrained to 110 nM according to Lin et al. (29), and these parameters were kept constant during fitting. Parameter values are presented in *SI Appendix*, Table S1. To quantify the “goodness of fit,” the rmsd was calculated for each fit.

EPSC amplitudes were converted to quantal content, the number of released SVs, by dividing the peak amplitudes by the estimated quantal size. For the PC–FSIN synapse (mean peak amplitude 160 pA), the quantal size is 32 pA (39), resulting in an initial release of 5 quanta. The quantal content of the PC–O-LM connection was estimated to be 1/10th of that of the PC-FSIN connection (20). Therefore, traces were scaled for an initial release of 0.5 quanta.

### Parameter Optimization for the PC–O-LM Connections.

First, we searched for a single parameter that would change the model from STD to STF. Results showed that only three parameters were capable of converting the model from STD to STF: b2, the backward rate constant of the second priming step, k2_0, the resting value of its forward rate constant, and P_fusion_ (*SI Appendix*, Table S1). While the model regimes exhibit STF, none of them describe adequately the data. Hence, we continued our search for model parameters that converted STD to STF, but this time changing two parameters simultaneously. Results showed that, while the two-parameter optimization was better than the one-parameter optimization, there were still great mismatches between the model and data. For example, the solution involving k2_0 and the steepness of its Ca^2+^ dependence, s2, had a reasonably good fit of the first release and the preconditioning EPSC amplitude train, but vastly underestimated the EPSC recovery; the P_fusion_+b2 solution produced an acceptable EPSC recovery but started with an initial SV release of zero. Allowing the simultaneous optimization of three parameters revealed parameter constellation that qualitatively described the dynamics of SV release at the PC–O-LM synapse. As shown in Fig. 4*A*, when k2_0, s2, and P_fusion_ were simultaneously optimized, the model qualitatively described the initial small facilitation and depression, followed by the large facilitation and depression during the high-frequency EPSC train. Furthermore, the recovery was also reasonably well described, reflected in a robust reduction of the rmsd value. Because k2_0 and s2 values were altered to a similar extent (>90% reduction compared to PC–FSIN values; *SI Appendix*, Table S1), we introduced a common scaling factor of these two parameters and refitted the data by allowing only changes in the scaling factor and P_fusion_. This fit resulted in an rmsd value (0.00057) almost identical to that obtained with separate fitting of k2_0 and s2 (0.00054; Fig. 4*B*). The rational to use a scaling factor for simultaneous modification of k2_0 and s2 is that their underlying molecular mechanisms might be biologically linked. Finally, we allowed all parameters to be optimized (with the exception of resting [Ca^2+^] and AP-induced [Ca^2+^] increments), which resulted in a further improvement of the goodness of fit (rmsd = 0.00027; Fig. 4*B*). Notably, the largest improvement involved the first EPSC response of the recovery train. All data are given as mean ± SD.

## Inclusion and Diversity.

We support inclusive, diverse, and equitable conduct of research.

## Supplementary Material

Appendix 01 (PDF)

## Data Availability

All raw and analyzed data have been deposited in Zenodo (55). All other data are included in the article and/or *SI Appendix*.
