# Supplementary material for: Different states of synaptic vesicle priming explain target cell type–dependent differences in neurotransmitter release
Source: Proc Natl Acad Sci U S A. 2024 Apr 24;121(18):e2322550121. doi: 10.1073/pnas.2322550121 (PMC11067035; doi:10.1073/pnas.2322550121)
Supplement: Supplementary file 1 — Appendix 01 (PDF) [file pnas.2322550121.sapp.pdf]

Suppl. TABLE 1: Parameter descriptions and values for the sequential two-step priming model related to data shown in Fig. 2, 4 & 5. Asterisks (*) indicate parameter values which have not been fitted but adopted from Lin et al. (29). In the 4-AP and PDBU fit, parameters indicated with ** have been kept the same as the O-LM control best fit parameters, apart from $\Delta[Ca^{2+}]$ which was constrained to 0.168 µM. Bold type indicates the different resting pool sizes determined by the model fitting.

		**PC – FSIN**			**PC – O-LM IN**			
Parameter abbreviation	Parameter description	Five protocols fit	Complex protocols & 5-Hz train fit	Mean of both	Control best fit	Control k2_0, s2 & P_{fusion} fit	4-AP best fit	PDBU best fit
P_{fusion}	SV fusion probability	0.62	0.58	0.60	0.33	*0.36*	0.85	0.29
k1_0	Rate constant for the ES → LS transition at rest (ms^{-1})	5.7E-04	6.5E-04	6.1E-04	7.1E-04	6.1E-04	2.1E-03	2.7E-03
b1	Rate constant for the ES ← LS transition (ms^{-1})	3.6E-04	4.0E-04	3.8E-04	1.3E-04	3.8E-04	1.0E-05	5.0E-06
k2_0	Rate constant for the LS → TS transition at rest (ms^{-1})	2.3E-04	2.5E-04	2.4E-04	8.2E-05	*2.2E-05*	1.0E-05	3.4E-04
b2	Rate constant for the LS ← TS transition (ms^{-1})	3.1E-04	2.9E-04	3.0E-04	1.0E-03	3.0E-04	2.0E-04	6.5E-04
κ	Fraction of LS transferred to TSL by an AP	0.20	0.17	0.18	0.19	0.18	0.18	0.49
b3	Decay time constant of the TSL state (ms)	70	75	72.5	38.0	72.5	35.8	37.7
s1	Ca-dependence of 1st priming rate constant	0.1	0.14	0.12	0.074	0.12	0.07	0.03
s2	Ca-dependence of 2nd priming rate constant	0.25	0.25	0.25	0.024	*0.012*	0.040	0.10
k_1	Michaelis-Menten K_D of 1st priming step (µM)	0.40	0.25	0.33	0.65	0.33	0.65**	0.59
N_{Tot}	Total number of release sites	26	25	25.5	24.1	25.5	27.8	31.0
$\Delta[Ca^{2+}]$	$[Ca^{2+}]$ increment per AP-induced 'effective' $[Ca^{2+}]$ transient (µM)	0.11*	0.11*	0.11*	0.11*	0.11*	0.168**	0.11**
$[Ca^{2+}]$ decay	Decay time constant of the AP-induced 'effective' $[Ca^{2+}]$ transient (ms)	95	110	102.5	65.3	102.5	81.8	65.3**
$[Ca^{2+}]$ rest	Basal $[Ca^{2+}]$ at rest (µM)	0.05*	0.05*	0.05*	0.05*	0.05*	0.05**	0.05**
RMSD	*Root-mean-square deviation*	*0.00100*	*0.00199*		*0.00027*	*0.00054*	*0.00060*	*0.00088*
**TS (rest)**	**'Tightly docked state' of an SV which is fusion-competent**			**8.42**	**1.57**	**1.1**	**1.34**	**10.58**
**LS (rest)**	**'Loosely docked state' of an SV which is not fusion-competent**			**10.52**	**19.11**	**15.04**	**26.37**	**20.38**
**ES (rest)**	**Empty state of a release site**			**6.56**	**3.46**	**9.4**	**0.13**	**0.04**
**TS fraction**	**Fraction of SVs in state TS state (TS/(LS+TS))**			**0.44**	**0.08**	**0.07**	**0.05**	**0.34**
